# Engineering of Immunoglobulin Fc Heterodimers Using Yeast Surface-Displayed Combinatorial Fc Library Screening

**DOI:** 10.1371/journal.pone.0145349

**Published:** 2015-12-16

**Authors:** Hye-Ji Choi, Ye-Jin Kim, Dong-Ki Choi, Yong-Sung Kim

**Affiliations:** Department of Molecular Science and Technology, Ajou University, Suwon 16499, Korea; Alexion Pharmaceuticals, UNITED STATES

## Abstract

Immunoglobulin Fc heterodimers, which are useful scaffolds for the generation of bispecific antibodies, have been mostly generated through structure-based rational design methods that introduce asymmetric mutations into the CH3 homodimeric interface to favor heterodimeric Fc formation. Here, we report an approach to generate heterodimeric Fc variants through directed evolution combined with yeast surface display. We developed a combinatorial heterodimeric Fc library display system by mating two haploid yeast cell lines, one haploid cell line displayed an Fc chain library (displayed Fc_CH3A_) with mutations in one CH3 domain (CH3A) on the yeast cell surface, and the other cell line secreted an Fc chain library (secreted Fc_CH3B_) with mutations in the other CH3 domain (CH3B). In the mated cells, secreted Fc_CH3B_ is displayed on the cell surface through heterodimerization with the displayed Fc_CH3A_, the detection of which enabled us to screen the library for heterodimeric Fc variants. We constructed combinatorial heterodimeric Fc libraries with simultaneous mutations in the homodimer-favoring electrostatic interaction pairs K370-E357/S364 or D399-K392/K409 at the CH3 domain interface. High-throughput screening of the libraries using flow cytometry yielded heterodimeric Fc variants with heterodimer-favoring CH3 domain interface mutation pairs, some of them showed high heterodimerization yields (~80–90%) with previously unidentified CH3 domain interface mutation pairs, such as hydrogen bonds and cation-π interactions. Our study provides a new approach for engineering Fc heterodimers that could be used to engineer other heterodimeric protein-protein interactions through directed evolution combined with yeast surface display.

## Introduction

Immunoglobulin G (IgG) is a monospecific, bivalent antigen-binding antibody consisting of two identical heavy chains and two identical light chains. Its assembly is driven by homodimerization of the fragment crystallizable (Fc) regions of the heavy chains and disulfide linkages between each heavy chain and each light chain [1]. Fc homodimerization of the heavy chains is initially driven by noncovalent inter-CH3 domain interactions and subsequently by disulfide linkages in the hinge region [2]. Replacement of the homodimer-favoring interactions at the CH3 domain interface with heterodimer-favoring interactions can generate Fc heterodimers, which can be used as scaffolds for IgG-like bispecific antibodies [3, 4]. Bispecific antibodies simultaneously bind to two different target antigens within a single molecule. Such bispecific antibodies have potential clinical benefits for the treatment of complicated diseases, such as tumors and immune disorders [5, 6], and more than 50 different bispecific antibodies have been reported [3, 4]. Among them, the heterodimeric Fc-based IgG-like format is attractive because it can be designed as close as possible to the natural IgG architecture such that it possesses desirable physicochemical properties, such as high stability, large-scale manufacturing capability, and low immunogenicity, in addition to the natural IgG-like properties of a long serum half-life and immune cell-recruiting effector functions [3, 4, 6].

Fc homodimerization is driven by both hydrophobic interactions at the center of the CH3 interface and symmetric electrostatic interactions around the rim of the hydrophobic core [7, 8]. Accordingly, most strategies used to generate heterodimeric Fc variants are structure-guided rational designs that introduce asymmetric mutations into the CH3 homodimeric interface to favor heterodimeric Fc formation [9]. In a pioneering approach, the so called “Knobs-into-holes (KiH)” Fc variant was generated, which has a T366W_CH3A_ “knob” mutation (EU numbering [10]) in one CH3 domain (CH3A) and T366S/L368A/Y407V_CH3B_ “hole” mutations in the other CH3 domain (CH3B) [11, 12]. These mutations created asymmetric steric complementarity at the core of the CH3 interface favoring Fc heterodimerization through hydrophobic interactions [13]. Similar strategies have been used to generate other heterodimeric Fc variants with sterically complementary mutations, including HA-TF [14], ZW1 [15], and SEEDBody [16]. Other approaches have generated the DD-KK [7] and EEE-RRR [17] heterodimeric Fc variants, in which the residues involved in the symmetric electrostatic interactions at the CH3 interface were replaced with residues that form asymmetric electrostatic interactions. Another structure-based design generated a heterodimeric Fc variant, EW-RVT, with two pairs of heterodimer-favoring interactions, K409W_CH3A_-D399V/F405T_CH3B_ (called the W-VT pair) and K360E_CH3A_-Q347R_CH3B_ (called the E-R pair), which were designed to replace the conserved electrostatic interactions with asymmetric hydrophobic interactions and to add asymmetric long-range electrostatic interactions at the rim of the heterodimeric CH3 interface, respectively [8, 18].

In this study, we aimed to generate heterodimeric Fc variants using a directed evolution approach combined with high-throughput screening. We reasoned that a directed evolution approach could be used to isolate novel, stable, heterodimeric Fc variants with high heterodimerization yields by introducing novel mutation pairs at the CH3 interface. For screening, we developed a semi-quantitative monitoring system for heterodimeric Fc formation using a yeast cell surface display technique combined with yeast mating [19, 20], which enabled the construction of a combinatorial heterodimeric Fc library on the yeast cell surface and screening using fluorescence-activated cell sorting (FACS). We isolated heterodimeric Fc variants with high heterodimerization yields (~80–90%) and previously unidentified CH3 domain interface interactions, including hydrogen bonds and cation-π interactions.

## Material and Methods

### Yeast strains and media

*Saccharomyces cerevisiae* strains JAR200 (MATa) and YVH10 (MATα) have been previously described in detail [20–22]. The composition of standard yeast media YPD, SDCAA, and SGCAA have been previously described in detail [20–22]. All of the reagents used were of analytical grade.

### Construction of yeast surface display and secretion vectors for Fc chain expression

To construct the yeast surface display vector for the Fc chain, the human IgG1 Fc gene carrying either the wild type CH3 domain or the variant CH3A sequence was subcloned in-frame into the *Nhe*I/*Bam*HI sites of the pCTCON yeast surface display vector (with a *TRP1* marker) [19, 23], generating pCTCON-displayed Fc_CH3A_. pCTCON-displayed Fc_CH3A_ expresses the Aga2 protein, the Fc protein, including the hinge-CH2-CH3 region (residues 225–447, EU number), and a Myc tag under the control of the *GAL1/10* promoter (S1 Fig). To construct the plasmid carrying the Fc chain for secretion with either the wild type CH3 domain or the CH3B variant, we subcloned the Fc variant in-frame using *Eag*I/*Afl*II into pRS316 with a *URA3* marker (Invitrogen) [20], generating pSEC2-secreted Fc_CH3B_ (S1 Fig). pSEC2-secreted Fc_CH3B_ expresses α-factor secretion signal peptide, the Fc region, and a Flag tag under the control of the *GAL1* promoter. In both plasmids, the bottom hinge region sequence (THTCPPCP) of Fc was modified by substituting Ser for Cys (THTSPPSP) to prohibit Fc homodimerization by disulfide bonding. Further, the N-glycosylation site Asn297 in the Fc region was changed to Gln (N297Q) to exclude hypermannosylation.

### Construction of the Fc gene libraries on yeast haploid cells

The Fc gene libraries were constructed by serial overlapping polymerase chain reaction (PCR) with degenerative primers designed to introduce mutations at the residues targeted in CH3A and CH3B, generating the displayed Fc_CH3A_ and secreted Fc_CH3B_ libraries, respectively. The primers used to construct the LibA1, LibB1, LibA2, and LibB2 libraries are listed in the S2 Table. The targeted residues were randomized using the degenerate codons DNB (D = A/G/T, N = A/C/G/T, B = C/G/T) in LibA1 and LibB1 and NNK (N = A/C/G/T, K = G/T) in LibA2 and LibB2. DNB encodes 17 amino acids (55.6% are nonpolar [Gly, Ala, Val, Leu, Ile, Phe, Tyr, Trp, Cys, Met], 27.8% are polar/uncharged [Ser, Thr, Asn], and 13.9% are charged [Asp, Glu, Lys, Arg]) and one stop codon [2.8%] (Pro, His, and Gln are excluded). NNK encodes all 20 amino acids (53.1% are nonpolar [Gly, Ala, Val, Leu, Ile, Pro, Phe, Tyr, Trp, Cys, Met], 21.9% are polar/uncharged [Ser, Thr, Asn, Gln], and 21.9% are charged [Asp, Glu, His, Lys, Arg]) as well as one stop codon (3.1%).

The displayed Fc_CH3A_ gene library (5 μg) and the pCTCON-displayed Fc vector linearized by *Nhe*I/*Bam*HI digestion (1 μg) were co-transformed into the JAR200 strain (MATa) by homologous recombination using a Bio-Rad Gene Pulser electroporation apparatus [20, 21] according to the improved yeast transformation method [24]. Likewise, the secreted Fc_CH3B_ gene library (5 μg) and the linearized pSEC2-secreted Fc vector (by *Eag*I/*Afl*II digestion, 1 μg) were co-transformed into the YVH10 strain (MATα). The transformants were plated directly on selective media; displayed Fc_CH3A_ library-transformed JAR200 cells were plated on SDCAA+Ura and secreted Fc_CH3B_ library-transformed YVH10 cells were plated on SDCAA+Trp [20, 21].

### Construction of a combinatorial heterodimeric Fc library using yeast mating and screening

The two haploid yeast cell lines carrying the displayed Fc_CH3A_ and secreted Fc_CH3B_ libraries were mated to obtain diploid cells carrying the combinatorial heterodimeric Fc library according to the optimized yeast mating protocol [20]. The library size was determined by plating serial 10-fold dilutions of the mated cells on selective SDCAA agar plates [20]. Diploid yeast cells carrying the library were grown in SDCAA at 30°C overnight, transferred (at OD_600_ = 0.5) to the induction medium (SGCAA) and grown at 30°C for 24 h in a shaking incubator set at 250 rpm. To label the surface-anchored secreted Fc_CH3B_ and heterodimeric displayed Fc_CH3A_-secreted Fc_CH3B_ assembly on the cell surface, cells were incubated with an anti-Flag mouse mAb and then with a secondary phycoerythrin (PE)-labeled anti-mouse mAb (sc-3738; Santa Cruz Biotechnology) and a fluorescein isothiocyanate (FITC)-labeled anti-human Fc goat mAb (F-9512; Sigma) prior to flow cytometric analysis. For the library screening, four rounds of FACS analysis were carried out using a FACS Aria II (Becton Dickinson, USA). During FACS, cells were fluorescence labeled using an anti-Flag mouse mAb and then a secondary PE-labeled anti-mouse mAb to sort cells with higher levels of surface-displayed secreted Fc_CH3B_. The final sorted yeast cells were plated on selective medium, and approximately 40 individual clones were randomly chosen and analyzed to obtain cells expressing high-yield heterodimeric Fc variants. The sequences of the heterodimeric Fc variants were determined by yeast colony PCR [20, 21].

### Evaluation of heterodimeric Fc yield in mammalian cells

The isolated Fc_CH3A_ and Fc_CH3B_ variant genes were subcloned in frame into the pcDNA3.1-scFv-Fc_CH3A_ and pcDNA3.1-Fc_CH3B_ vectors to express scFv-Fc_CH3A_ (hAY4 scFv-hinge-CH2-CH3A) and Fc_CH3B_ (hinge-CH2-CH3B), respectively, in mammalian cells [8, 18]. To coexpress scFv-Fc_CH3A_/Fc_CH3B_, the two plasmids encoding scFv-Fc_CH3A_ and Fc_CH3B_ were transiently cotransfected at the indicated molar ratios with polyethylenimine (25-kDa; Polyscience) into 30–200 mL of HEK293F cells in FreeStyle 293 media (Invitrogen) as described previously [8]. After 6 days of culture, the proteins were purified from the culture supernatants using Protein-A agarose chromatography (GE Healthcare, Uppsala, Sweden) [25]. The purified proteins were analyzed by SDS-PAGE to estimate the heterodimerization yields as described previously [8, 18].

### Expression and characterization of the heterodimeric Fc_CH3A_/Fc_CH3B_ proteins

Fc_CH3A_ variant genes were subcloned into pcDNA3.1-Fc to generate pcDNA3.1-Fc_CH3A_. To coexpress Fc_CH3A_/Fc_CH3B_, the two plasmids, pcDNA3.1-Fc_CH3A_ and pcDNA3.1-Fc_CH3B_, were transiently cotransfected at an equivalent molar ratio into HEK293F cells as described above [8, 18]. The Fc_CH3A_/Fc_CH3B_ proteins were purified as described above [25], and the purified proteins were analyzed by SEC, DSC, and SPR to determine pH-dependent FcRn binding as previously described [8, 18, 25].

### Statistical analysis

Data are reported as mean ± SD of at least 3 independent experiments performed in triplicate, unless otherwise specified. Statistical significance was analyzed by a 2-tailed unpaired Student’s *t*-test using Excel (Microsoft, Inc.). A *P* value less than 0.05 was considered statistically significant.

## Results

### Design and establishment of a heterodimeric Fc monitoring system using yeast cell surface display

**Fig 1:**
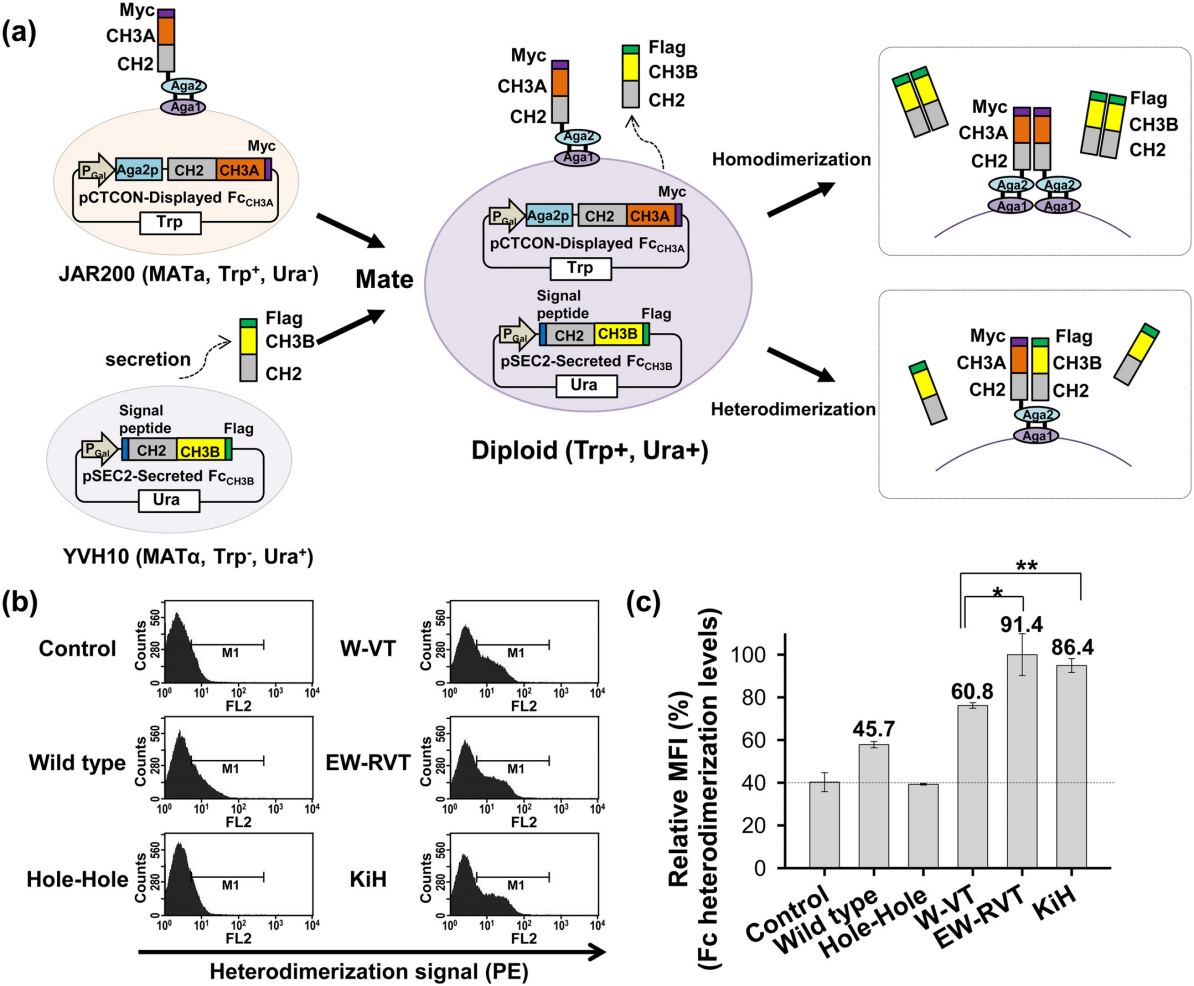
Design and validation of the heterodimeric Fc yeast surface display system. (a) Overall strategy for Fc heterodimer display on the yeast cell surface. Two haploid cell lines were transformed; JAR200 (MATa) displayed one Fc chain (displayed Fc_CH3A_) via fusion to Aga2 on the cell surface and YVH10 (MATα) secreted the other Fc chain (secreted Fc_CH3B_), respectively. Then, the two haploid cell lines were mated to generate diploid cells that coexpress displayed Fc_CH3A_ and secreted Fc_CH3B_. If either the displayed Fc_CH3A_ or the secreted Fc_CH3B_ favors homodimerization, the secreted Fc_CH3B_ will be released into the extracellular space (*top right*). If secreted Fc_CH3B_ favors heterodimerization with the displayed Fc_CH3A_, it will be anchored to the cell surface due to its binding with the displayed Fc_CH3A_ (*right bottom*). The levels of Fc heterodimerization on the cell surface can be monitored by immunofluorescence labeling of the C-terminal Flag tag on secreted Fc_CH3B_. (b) Histogram showing the levels of surface-displayed secreted Fc_CH3B_ (i.e., Fc heterodimerization levels) on diploid cells coexpressing the indicated Fc variant pairs or wild-type Fc pair, as determined by flow cytometry after labeling with an anti-Flag mouse mAb and a secondary phycoerythrin (PE)-labeled anti-mouse mAb (*x*-axis). (c) Semi-quantitative comparisons of Fc heterodimerization levels as a percentage of the MFI of the Fc variants compared with that of the EW-RVT Fc variant (set as 100%). Error bars, ± SD of 3 independent experiments. * *P* < 0.05 and **P < 0.01. The numbers above the bar indicate the previously reported heterodimerization yields of the Fc variants. In (b and c), the control is cells labeled with only the secondary PE-conjugated antibody, and the relative MFI is shown as a dotted line in (c) to indicate the background MFI with negligible cell surface-displayed levels of secreted Fc_CH3B_.

First, we developed a novel monitoring system for the formation of Fc heterodimers on the yeast cell surface to quantitatively measure Fc heterodimerization by coexpressing one Fc chain that was designed to be anchored to the cell wall and a second Fc chain that was designed to be secreted (Fig 1a). Our strategy was to display an Fc variant (called displayed Fc_CH3A_) with a CH3 domain (CH3A) on the cell surface of one haploid cell line and secrete the other Fc variant (called secreted Fc_CH3B_) with the other CH3 domain (CH3B) in another haploid yeast cell line. Then, mate the two haploid cell lines to generate diploid cells that coexpress the displayed Fc_CH3A_ and the secreted Fc_CH3B_. If the two Fc variants favor heterodimerization over homodimerization, during secretion, the secreted Fc_CH3B_ will be anchored on the cell surface through its heterodimeric interaction with the displayed Fc_CH3A_, such that the secreted Fc_CH3B_ can be detected on the yeast cell surface by immunofluorescence. However, if either the displayed Fc_CH3A_ or the secreted Fc_CH3B_ favors homodimerization, the secreted Fc_CH3B_ will be secreted to the culture supernatant without being displayed on the cell surface (Fig 1a).

To validate the above system, we used previously reported heterodimeric Fc variants of human IgG1 with different heterodimerization yields, such as W-VT (~61% heterodimerization yield) [8], EW-RVT (~91% yield) [8], and KiH (~86% yield) [11]. As controls, a wild-type (WT) Fc and a KiH hole-hole variant disfavoring heterodimerization were included. The Fc variant pairs, including the hinge-CH2-CH3 domains (residues 225–447, EU number), were subcloned into two plasmids: pCTCON-displayed Fc_CH3A_ for yeast cell surface display of one Fc variant (displayed Fc_CH3A_) and pSEC2-secreted Fc_CH3B_ for secretion of the other Fc variant (secreted Fc_CH3B_; Fig 1a and S1 Fig). A Flag tag was fused to the C-terminus of the secreted Fc_CH3B_ variants to monitor the amount displayed on the cell surface in an assembled format with displayed Fc_CH3A_ (Fig 1a). The Fc variants contained two mutations (C228S and C231S) in the hinge region to exclude Fc homodimerization by eliminating the natural disulfide bonds in the hinge region and an N297Q mutation to avoid yeast hypermannosylation by removing the N-linked glycosylation site in the CH2 domain. The pCTCON-displayed Fc_CH3A_ and pSEC2-secreted Fc_CH3B_ plasmids were transformed into JAR200 (MATa) and YVH10 (MATα) cells, respectively. The two transformed haploid cell lines were mated to generate diploid cells [20, 21], which were then treated to induce coexpression of the Fc variants. Flow cytometric analysis revealed that the diploid cells had a Flag tag fused to the C-terminus of secreted Fc_CH3B_ (Fig 1b). However, cells expressing the WT Fc and KiH hole-hole variants showed weak and negligible Flag tag-positive fluorescence, respectively. Semi-quantitative comparisons of the mean fluorescence intensity (MFI) showed a close correlation between Flag MFI and the previously reported heterodimerization yields of Fc variants, with higher MFI values for Fc variants with higher heterodimerization yields (Fig 1c). These results suggested that the strength of Fc heterodimerization is directly correlated with the amount of cell surface-displayed secreted Fc_CH3B_, allowing semi-quantitative monitoring of Fc heterodimerization on the yeast cell surface.

### Design and construction of combinatorial heterodimeric Fc libraries

**Fig 2:**
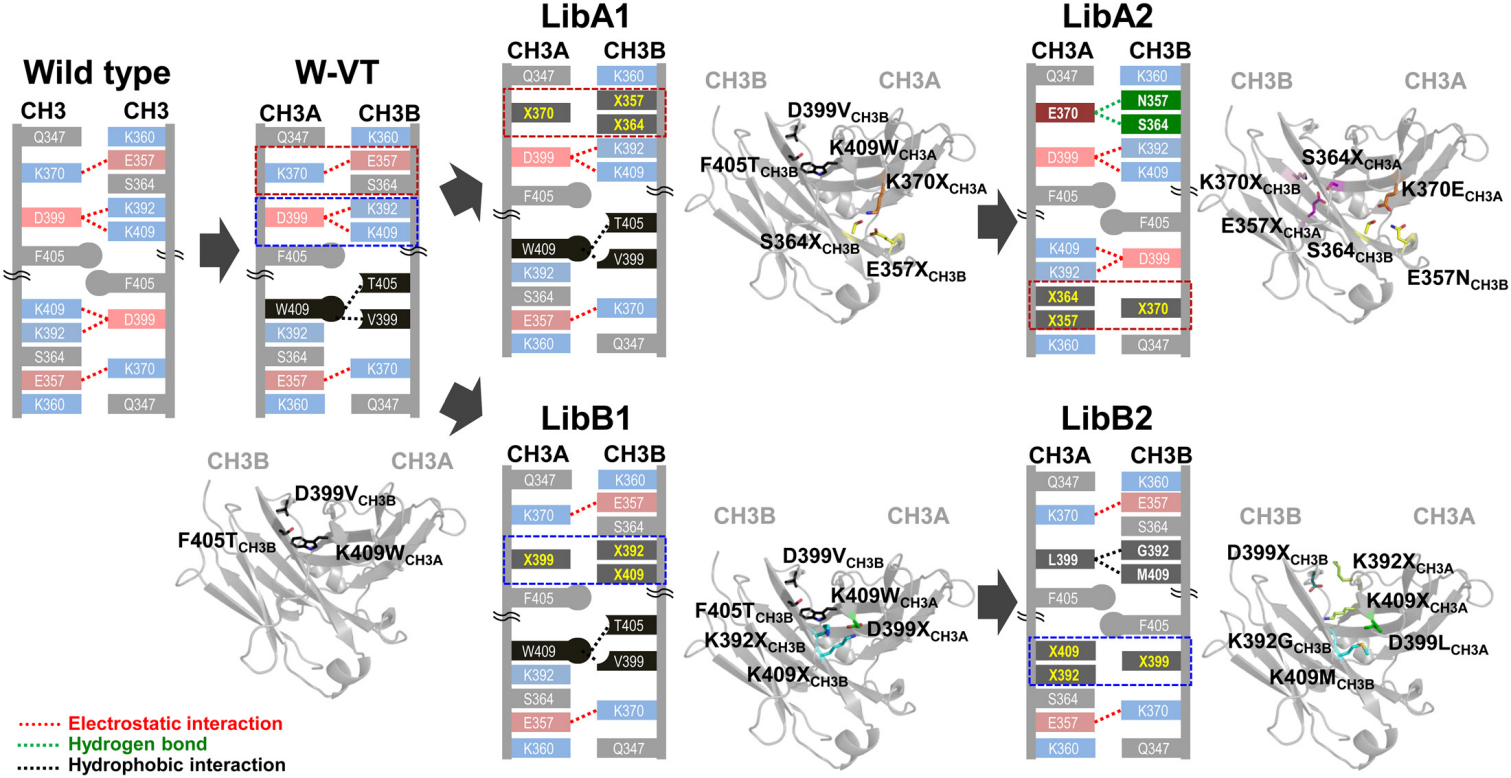
Schematic diagrams showing the overall strategy and sequential process of constructing the combinatorial heterodimeric Fc library. The cartoons depict major interactions contributing to the homodimeric CH3 interactions of wild type Fc and the heterodimeric CH3A-CH3B interactions in the heterodimeric Fc variants. The first libraries (LibA1 and LibB1) were constructed based on the W-VT variant (with K409W_CH3A_-D399V/F405T_CH3B_) by randomizing the homodimer-favoring K370_CH3A_-E357/S364_CH3B_ and D399_CH3A_-K392/K409_CH3B_ paired residues, respectively, which are located in the opposite side of the W-VT variant pair. Screening of the LibA1 and LibB1 libraries generated the variants with the highest heterodimerization yield, A107_w/o W-VT_ (K370E_CH3A_-E357N_CH3B_) and B168_w/o W-VT_ (D399L_CH3A_-K392G/K409M_CH3B_), respectively. In the LibA2 and LibB2 libraries, the other homodimer-favoring residues E357/S364_CH3A_-K370_CH3B_ and K392/K409_CH3A_-D399_CH3B_, which are located on the opposite site of the mutated residues in LibA1 and LibB1, were randomized based on the A107_w/o W-VT_ and B168_w/o W-VT_ templates, respectively. The CH3 domain interfaces were modeled using the 2.5 Å crystal structure of the EW-RVT variant (PDB code 4X98; [18]). The mutant residues in each library are depicted as sticks and indicated as X. The images were illustrated using PyMOL (Schrödinger, Inc.).

Using our Fc heterodimer display and semi-quantitative assay system, we sought to construct a combinatorial heterodimeric Fc library with CH3 interface mutations and screen it to isolate novel heterodimeric Fc variants. Homodimerization of WT Fc is mainly driven by core hydrophobic interactions (involving L351, T366, L368, and Y407) at the CH3 interface and two paired electrostatic interactions (K370-E357/S364 and D399-K392/K409) at the first and second shell of the CH3 interface around the rim of the hydrophobic core (Fig 2) [7, 8]. In this study, we mutated the two homodimer-favoring electrostatic interaction pairs while conserving the hydrophobic core interactions at the CH3 interface because we reasoned that the hydrophobic core interactions would also be important for the thermodynamic stability of the Fc heterodimer. The two targeted electrostatic interaction pairs are symmetrically duplicated on both sides of the hydrophobic core due to the 2-fold symmetry interface of the CH3 homodimer (Fig 2).

We recently reported a W-VT Fc variant with ~61% heterodimerization yield (Table 1) [8]. To improve the heterodimerization yield, we first designed two separate libraries by respectively randomizing the paired K370_CH3A_-E357/S364_CH3B_ and D399_CH3A_-K392/K409_CH3B_ residues, which are located on the opposite side of the W-VT variant pair K409W_CH3A_-D399V/F405T_CH3B_ (Fig 2). Based on the W-VT variant template, we constructed two heterodimeric Fc libraries with randomized mutations at three residues, K370_CH3A_, E357_CH3B_, and S364_CH3B_, in one library (called LibA1) and D399_CH3A_, K392_CH3B_, and K409_CH3B_ in another library (called LibB1) with the degenerate codon DNB (D = A/G/T, N = A/C/G/T, B = C/G/T; Fig 2). The DNB codon, encoding 17 amino acids with preference of nonpolar (55.6%) and polar (27.8%) amino acids to charged amino acids (13.9%), was chosen to possibly substitute the original symmetric electrostatic interactions with the other asymmetric noncovalent interactions. A displayed Fc_CH3A_ library with the aforementioned CH3A mutations and a secreted Fc_CH3B_ library with the CH3B mutations were constructed in the haploid yeast strains JAR200 (MATa) and YVH10 (MATα), respectively. The two haploid cell lines were mated to obtain diploid cells carrying the combinatorial heterodimeric Fc libraries LibA1 and LibB1. The diversity of LibA1 (~3.0 × 10^7^) and LibB1 (~2.8 × 10^7^) covered the respective theoretical library size of ~5 × 10^3^, estimated by 17×17×17 = 4913.

**Table 1:** Summary of the mutations, heterodimerization yields, production yields, and heterodimer-favoring and homodimer-disfavoring interactions of the isolated heterodimeric Fc variants from LibA libraries

	Paired mutations ^a^		Yield (scFv-Fc_CH3A_/Fc_CH3B_ system)		Main interactions ^d^		
Variant	CH3A chain	CH3B chain	Heterodimer (%) ^b^	Production (%) ^c^	Favoring CH3A-CH3B	Disfavoring CH3A-CH3A	Disfavoring CH3B-CH3B
**W-VT**	K409W	D399V/F405T	60.8 ± 3.0	102 ± 31.1	K409W_CH3A_-D399V/F405T_CH3B_ complementary hydrophobic interaction	F405_CH3A_-K409W_CH3A_ steric clash	K392E_CH3B_ unpaired charged residue K409_CH3B_ unpaired charged residue
**Variants from the LibA1 library constructed using a W-VT template with mutation pairs K409W**_**CH3A**_ **-D399V**_**CH3B**_ **/F405T**_**CH3B**_							
**A107**	**K370E**/K409W	**E357N**/D399V/F405T	93.4 ± 1.1 (78.2 ± 4.2)	108 ± 51.1 (103 ± 49.0)	K370E_CH3A_-E357N_CH3B_ hydrogen bond K370E_CH3A_- S364_CH3B_ hydrogen bond Y349_CH3A_-E357N_CH3B_ hydrogen bond K370E_CH3B_-K409_CH3B_ electrostatic interaction	E357_CH3A_-K370E_CH3A_ electrostatic repulsion	–
**A108**	**K370E**/K409W	**E357I/S364T**/D399V/F405T	70.5 ± 3.3	125 ± 78.1	K370E_CH3A_-K409_CH3B_ electrostatic interaction	E357_CH3A_-K370E_CH3A_ electrostatic repulsion	–
**A109**	**K370M**/K409W	**E357M/S364W**/D399V/F405T	90.5 ± 2.7 (61.6 ± 4.5)	102 ± 27.2 (86.9 ± 2.1)	K370M_CH3A_-E357M/S364W_CH3B_ complementary hydrophobic interaction	E357_CH3A_ unpaired charged residue	K370_CH3B_-S364W_CH3B_ steric clash
**A146**	**K370D**/K409W	**E357M**/D399V/F405T	74.5 ± 3.4 (71.0 ± 3.7)^d^	127 ±22.7 (89.5 ± 13.5)	K370D_CH3A_-K409_CH3B_ electrostatic interaction K370D_CH3A_-S364_CH3B_ hydrogen bond	K370D_CH3A_-E357_CH3A_ electrostatic repulsion	–
**Variants from the LibA2 library constructed using a A107**_**w/oW-VT**_ **template with mutation pairs K370E**_**CH3A**_ **-E357N**_**CH3B**_							
**A205**	**E357D/S364W**/K370E	E357N/**K370R**	88.8 ± 2.2	118 ± 5.0	S364W_CH3A_-K370R_CH3B_ cation-π	E357D_CH3A_-S364W_CH3A_ anion-π repulsion K370E_CH3A_-E357D_CH3A_ electrostatic repulsion	Hole-hole interface
**A210**	**E357A/S364Y**/K370E	E357N/**K370H**	80.8 ± 5.8	104 ± 9.2	S364Y_CH3A_-K370H_CH3B_ π-π, S364Y_CH3A_-K370H_CH3B_ hydrogen bond	S364Y_CH3A_-K370E_CH3A_ anion-π repulsion	Hole-hole interface
**A216**	**E357G/S364W**/K370E	E357N	80.3 ± 4.6	92.1 ± 13.1	S364W_CH3A_-K370_CH3B_ cation-π	S364W_CH3A_-K370E_CH3A_ anion-π repulsion	–
**A241**	**E357N/S364W**/K370E	E357N	81.0 ± 3.9	150 ± 49.6	S364W_CH3A_-K370_CH3B_ cation-π	S364W_CH3A_-K370E_CH3A_ anion-π repulsion	–

^a^ Newly introduced mutations in the CH3A or CH3B domain of the isolated Fc variants are highlighted in bold. Other mutations were present in the template variant.

^b^ Heterodimer yield (mean ± SD of three independent experiments) was determined by SDS-PAGE analyses under non-reducing conditions of the purified proteins after coexpression of the scFv-Fc_CH3A_/Fc_CH3B_ proteins carrying the indicated CH3 variant pair in HEK293F cells as described in the text.

^c^ The values represent relative purification levels (mean ± SD of three independent experiments) of Fc variants from HEK923 cells coexpressing scFv-Fc_CH3A_/Fc_CH3B_ proteins for 6 days compared with the purification yield of the EW-RVT variant (3.1 ± 0.7 mg/100 mL culture).

In (^b^ and ^c^), the values in parenthesis are the heterodimerization and purification yields of the A107_w/o W-VT_, A109_w/o W-VT_, A146_w/o W-VT_, and B168_w/o W-VT_ Fc variants, in which the W-VT mutation pairs were back-mutated to the corresponding wild-type residues.

^d^ The heterodimer-favoring and homodimer-disfavoring interactions are those involving the newly introduced mutations on the CH3A or CH3B domain of the isolated heterodimeric Fc variants. Thus, the heterodimer-favoring and homodimer-disfavoring interactions from the parent template Fc variant should also be considered. The hole-hole interface means the absence of favorable intermolecular interactions at the CH3 domain interfaces due to less packing of amino acids with small-sized side chains. The minus “-” means that there are no particular repulsive interactions disfavoring CH3B-CH3B homodimerization, except for loss of the wild-type homodimer-favoring electrostatic interactions.

### Screening and isolation of heterodimeric Fc variants

**Fig 3:**
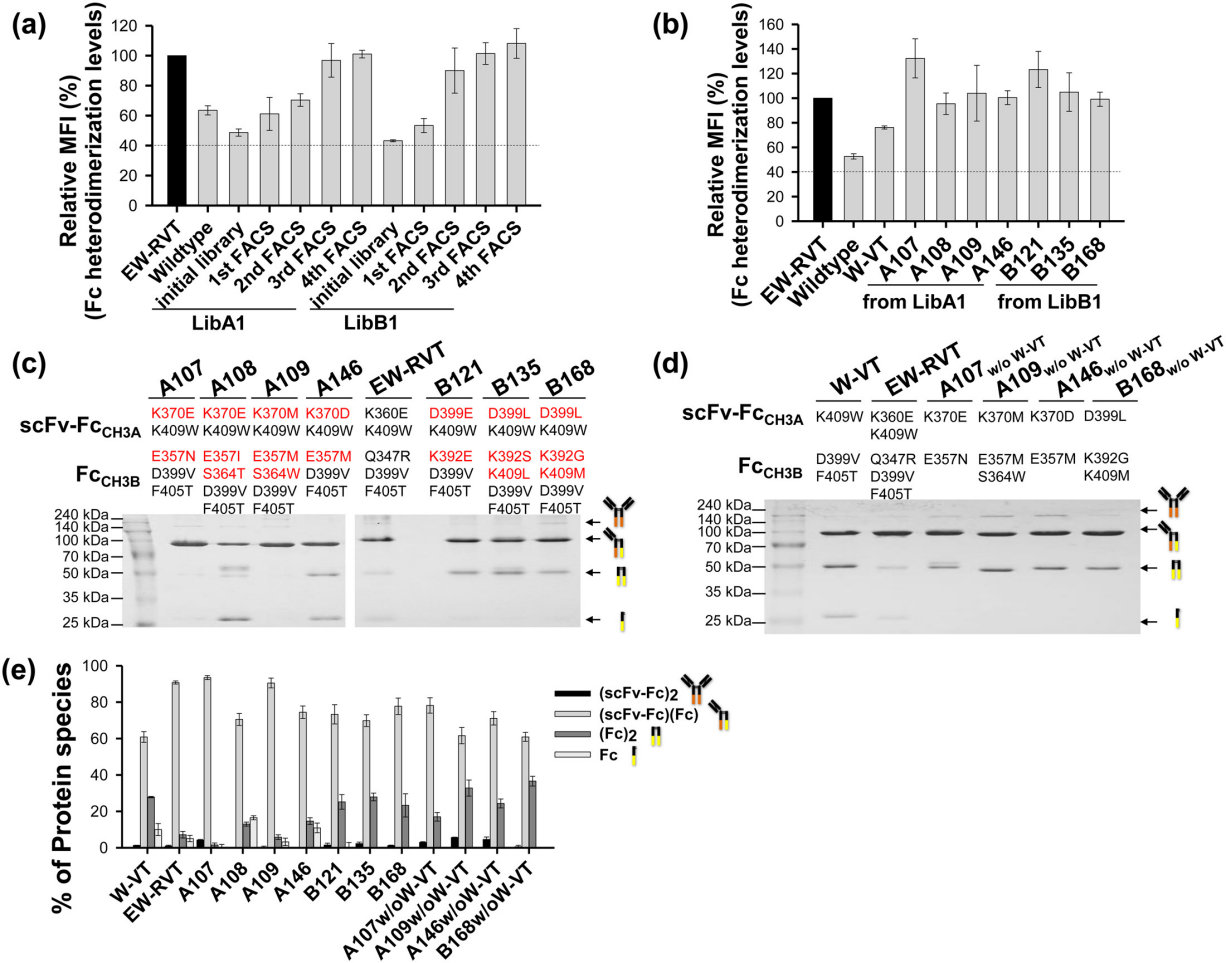
Isolation and characterization of heterodimeric Fc variants from the LibA1 and LibB1 libraries. (a) Enrichment profiles of diploid cells with higher Fc heterodimerization levels during the four sequential rounds of FACS with each library, indicated by the relative MFI (%) of each sample compared with that of the EW-RVT Fc variant (set as 100%). (b) Comparison of the Fc heterodimerization levels of the isolated Fc variants on the yeast cell surface shown as a percentage of the MFI relative to that of the EW-RVT Fc variant (set as 100%). In (a and b), the dotted baseline is the background MFI, which had negligible cell surface-displayed levels of secreted Fc_CH3B_. (c and d) SDS-PAGE analysis under non-reducing conditions of the purified coexpressed scFv-Fc_CH3A_/Fc_CH3B_ proteins (5 μg) carrying the indicated CH3 variant pairs. The newly introduced mutations in the CH3A or CH3B domain are shown in red font. The W-VT and EW-RVT Fc variants were included for comparison. The arrows indicate the assembled scFv-Fc_CH3A_ homodimer (~150 kDa), scFv-Fc_CH3A_/Fc_CH3B_ heterodimer (~78 kDa), Fc_CH3B_ homodimer (~53 kDa), and unassembled Fc_CH3B_ monomeric species (~ 27 kDa). (e) Heterodimer yield of the indicated scFv-Fc_CH3A_/Fc_CH3B_ proteins. The yields of each protein were quantified by measuring the relative band intensity on non-reducing SDS-PAGE gels, as shown in (c and d), using ImageJ. Error bars, ± SD of three independent experiments.

Using the LibA1 and LibB1 libraries, we carried out four sequential rounds of FACS for each library after labeling cells with an immunofluorescent Flag tag to detect the secreted Fc_CH3B_ anchored on the cell surface through heterodimerization with the displayed Fc_CH3A_. Cells displaying high levels of the Fc heterodimer were gradually enriched, as shown by the increased MFI obtained by setting the same gate (Fig 3a). Analysis of the final 40 sorted individual cells showed four unique Fc variants (A107, A108, A109, and A146) from LibA1 and three unique Fc variants (B121, B135, and B168) from LibB1 (Tables 1 and 2). The isolated clones showed much higher surface-displayed levels of Fc heterodimer than the parent W-VT variant (Fig 3b), suggesting that our heterodimeric Fc library display and screening technique is useful for generating functional heterodimeric Fc variants.

**Table 2:** Summary of the mutations, heterodimerization yields, production yields, and heterodimer-favoring and homodimer-disfavoring interactions of the isolated heterodimeric Fc variants from LibB libraries.

	Paired mutations ^a^		Yield (scFv-Fc_CH3A_/Fc_CH3B_ system)		Main interactions ^d^		
Variant	CH3A chain	CH3B chain	Heterodimer (%) ^b^	Production (%) ^c^	Favoring CH3A-CH3B	Disfavoring CH3A-CH3A	Disfavoring CH3B-CH3B
**Variants from the LibB1 library constructed using the W-VT template with mutation pairs K409W**_**CH3A**_ **-D399V**_**CH3B**_ **/F405T**_**CH3B**_							
**B121**	**D399E**/K409W	**K392E**/D399V/F405T	73.3 ± 5.3	127 ± 29.3	D399E_CH3A_-K409_CH3B_ electrostatic interaction	D399E_CH3A_-K409W_CH3A_ anion-π repulsion	K392E_CH3B_ unpaired charged residue K409_CH3B_ unpaired charged residue
**B135**	**D399L**/K409W	**K392S**/**K409L**/D399V/F405T	67.8 ± 3.2	104 ± 31.4	D399L_CH3A_-K392S/K409L_CH3B_ complementary hydrophobic interaction	K392_CH3A_ unpaired charged residue	Hole-hole interface
**B168**	**D399L**/K409W	**K392G**/**K409M**/D399V/F405T	77.7 ± 4.5 (60.9 ± 2.4)	119 ± 24.8 (96.9 ± 18.4)	D399L_CH3A_-K392G/K409M_CH3B_ complementary hydrophobic interaction	K392_CH3A_ unpaired charge residue	Hole-hole interface
**Variants from the LibB2 library constructed using the B168**_**w/o W-VT**_ **template with mutation pairs D399L**_**CH3A**_ **-K392G**_**CH3B**_ **/K409M**_**CH3B**_							
**B212**	**K392I**/D399L	**D399G/**K392G/K409M	65.3 ± 4.3	123 ± 31.2	K409_CH3A_-F405_CH3B_ cation-π	K409_CH3A_ unpaired charged residue	Hole-hole interface
**B215**	**K392R**/**K409R**/D399L	**D399W**/K392G/K409M	78.1 ± 6.9	114 ± 12.9	K392R_CH3A_-D399W_CH3B_ cation-π K409R_CH3A_-D399W_CH3B_ cation-π	K392R_CH3A_ unpaired charged residue K409R_CH3A_ unpaired charged residue	K409M_CH3B_-D399W_CH3B_ steric clash
**B235**	**K392C**/D399L	**D399C**/K392G/K409M	89.4 ± 4.1	101 ± 25.3	K392C_CH3A_-D399C_CH3B_ disulfide bond	K409_CH3A_ unpaired charged residue	Hole-hole interface
**B239**	**K392L**/D399L	**D399S**/K392G/K409M	76.3 ± 6.8	93.8 ± 31.2	K409_CH3A_-D399S_CH3B_ hydrogen bond K409_CH3A_-F405_CH3B_ cation-π	K409_CH3A_ unpaired charged residue	Hole-hole interface
**B240**	**K392S**/**K409R**/D399L	**D399G**/K392G/K409M	83.3 ± 4.7	104 ± 16.9	K409R_CH3A_-F405_CH3B_ cation-π	K409R_CH3A_ unpaired charged residue	Hole-hole interface
**B256**	**K392N**/D399L	**D399V**/K392G/K409M	79.4 ± 5.2	103 ± 11.8	K409_CH3A_-F405_CH3B_ cation-π	K409_CH3A_ unpaired charged residue	Hole-hole interface

^a, b, c, d^ The legends are same with those of Table 1.

To determine heterodimerization yields using the scFv-Fc_CH3A_/Fc_CH3B_ system in mammalian cells [7, 8], the Fc_CH3A_ and Fc_CH3B_ chain of the isolated Fc variants were subcloned in-frame into pcDNA3.1-scFv-Fc_CH3A_ and pcDNA3.1-Fc_CH3B_ vectors for expression in the scFv-hinge-CH2-CH3A and hinge-CH2-CH3B formats, respectively [8, 18]. Coexpression of homodimer can be distinguished from heterodimer based on molecular mass in SDS-PAGE analysis, as shown in Fig 3c [7, 8]. The two plasmids were transiently cotransfected at equivalent molar ratios into HEK293F cells. When the purified proteins from culture supernatants were separated by SDS-PAGE under non-reducing conditions, the isolated Fc variants existed predominantly as assembled heterodimers with minor portions of two homodimers (Fig 3c). Quantification of the relative band intensity revealed that all of the isolated Fc variants exhibited higher heterodimerization yields (~68–93%) than the parent W-VT variant (~61%; Fig 3e and Tables 1 and 2). Notably, the A107 variant isolated from LibA1 showed the highest yield (~93%), which was higher than that of the previously reported best variant, EW-RVT (~91%) [8]. Among the three Fc variants from LibB1, the B168 variant showed the highest yield (~78%). When the A107 and B168 variants were tested, we found that heterodimer yield was sensitive to the cotransfection molar ratio of scFv-Fc_CH3A_ to Fc_CH3B_ (S2 Fig), as was previously observed with KiH and other variants [7, 8, 11], and the best yield was obtained at a 1:1 molar ratio.

The new Fc variants contained the heterodimeric Fc-favoring interaction pair from the parent W-VT variant, K409_CH3A_-D399_CH3B_/F405_CH3B_. We assessed the heterodimerization ability of the newly selected mutation pairs in the top four heterodimer-forming Fc variants (A107, A109, A146, and B168) by generating Fc variants without the W-VT pair, A107_w/o W-VT_ (K370E_CH3A_-E357N_CH3B_), A109_w/o W-VT_ (K370M _CH3A_-E357M/S364W_CH3B_), A146_w/o W-VT_ (K370D_CH3A_-E357M_CH3B_), and B168_w/o W-VT_ (D399L_CH3A_-K392G/K409M_CH3B_). When the heterodimerization yield was determined using the scFv-Fc_CH3A_/Fc_CH3B_ system (Fig 3d), all four variants showed greater than ~60% heterodimerization yield, and the A107_w/o W-VT_ variant showed the highest heterodimerization yield (~78%; Fig 3d and 3e; Tables 1 and 2). These results suggest that the newly isolated mutation pairs from the yeast surface-display combinatorial Fc libraries have the ability to induce Fc heterodimer formation independent of the parent W-VT pair.

### Structural analysis of the new heterodimeric Fc variants

**Fig 4:**
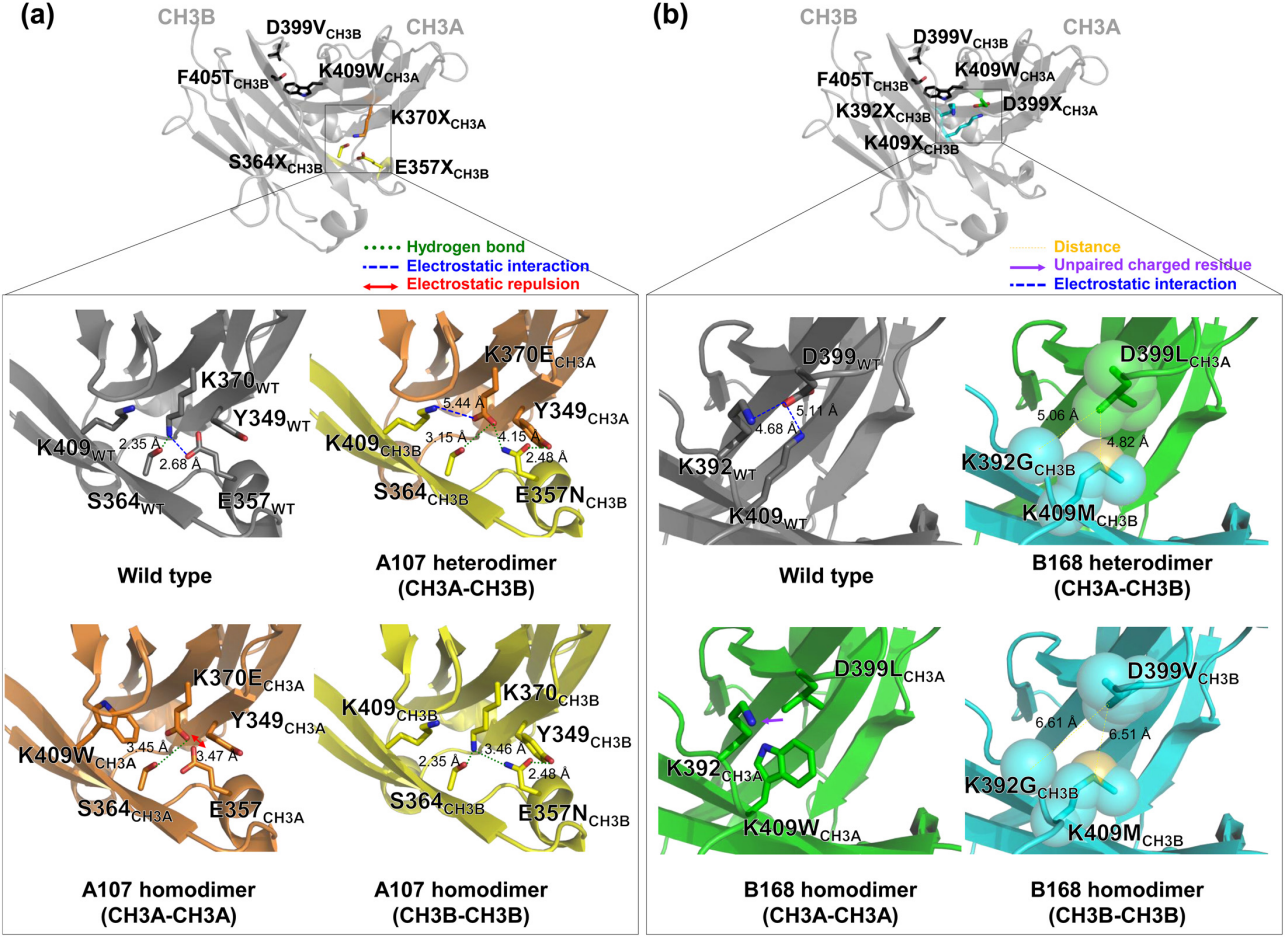
Modeled CH3 domain interface structures of the A107 (a) and B168 (b) variants based on the crystal structure of EW-RVT variant (PDB code 4X98). Each upper panel shows the CH3A-CH3B heterodimer structure of the W-VT variant to highlight the targeted mutation sites in LibA1 (a) and LibB1 (b) (as highlighted in the square) on the opposite side of the parent W-VT variant pair (K409W_CH3A_-D399V/F405T_CH3B_) at the CH3 interface. The lower panels shows a close-up view of the newly introduced mutation pair in A107 (K370E_CH3A_-E357N_CH3B_) (a) and B168 (D399L_CH3A_-K392G/K409M_CH3B_) (b) in a CH3A-CH3B heterodimer, CH3A-CH3A homodimer, and CH3B-CH3B homodimer, compared to the wild type CH3 homodimer. Details are described in the text.

To understand how the newly isolated CH3 mutation pairs favor Fc heterodimerization, we modeled the CH3 interfaces of the Fc variants based on the 2.5 Å crystal structure of the EW-RVT variant (PDB code 4X98; [18]). As described in detail in Tables 1 and 2, the newly introduced mutation pairs replaced the homodimer-favoring electrostatic interactions with CH3A-CH3B heterodimer-stabilizing interactions, such as hydrogen bonds (A107), electrostatic interactions (A108, A146, B121), and sterically complementary hydrophobic interactions (A109, B135, B168). CH3A-CH3A homodimer formation is likely disfavored largely due to repulsive electrostatic interactions (A107, A108, A146) and anion-π repulsive interactions (B121) as well as the presence of unpaired charged residues at the hydrophobic CH3 interface (A109, B135, B168). Similarly, CH3B-CH3B homodimer formation seems to be disfavored due to steric clash (A109), unpaired charged residues (B121), and the absence of stabilizing interactions at the less-packed CH3 interface (the so-called hole-hole interface; B135, B168; Table 2). For some variants (A107, A108, A146), there are no particular repulsive interactions disfavoring CH3B-CH3B homodimerization except for loss of the wild type homodimer-favoring electrostatic interactions (Fig 4a). This suggests that the heterodimerization of Fc variants is also more kinetically favorable than homodimer formation [8]. In particular, the A107 variant, which has the highest heterodimerization yield, seems to favor heterodimerization via hydrogen bonding between K370E_CH3A_-E357N, K370E_CH3A_-S364_CH3B_, and Y349_CH3A_-E357N in addition to the electrostatic interactions between K370E_CH3A_-K409_CH3B_ (Fig 4a). The CH3A-CH3A homodimer is most likely discouraged by the repulsive electrostatic interactions of E357_CH3A_-K370E_CH3A_ (Fig 4a). For the best variant from the LibB1 library, B168, the mutation pair D399L_CH3A_-K392G/K409M_CH3B_ likely drives heterodimerization via complementary hydrophobic interactions, while the unpaired charged residue K392_CH3A_ and the hole-hole interface of D399V_CH3B_-K392G/K409M_CH3B_ seem to discourage CH3A-CH3A and CH3B-CH3B homodimerization, respectively (Fig 4b).

### Isolation and characterization of heterodimeric Fc variants from the second-generation combinatorial heterodimeric Fc libraries

The two targeted electrostatic interaction pairs K370_CH3A_-E357/S364_CH3B_ and D399_CH3A_-K392/K409_CH3B_ in LibA1 and LibB1, respectively, are symmetrically duplicated on the opposite site of the CH3 interface on which the W-VT mutation pair was originally present (Fig 2). We sought to improve the heterodimerization yield of A107_w/o W-VT_ and B168_w/o W-VT_ by replacing other homodimer-favoring electrostatic interaction pairs (Fig 2). Using the heterodimeric Fc display system (Fig 1), we constructed a LibA2 library randomizing the E357/S364_CH3A_-K370_CH3B_ residues based on the A107_w/o W-VT_ variant (K370E_CH3A_-E357N_CH3B_) and a LibB2 library randomizing K392/K409_CH3A_-D399_CH3B_ based on the B168_w/o W-VT_ variant (D399L_CH3A_-K392G/K409M_CH3B_) using the degenerate codon NNK (N = A∕T∕G∕C, K = G∕T). The NNK codon encoding all 20 amino acids was adopted to maximally randomize the targeted residues. The diversity of the LibA2 (~3.3 × 10^7^) and LibB1 (~3.0 × 10^7^) libraries exceeded the theoretical library size of ~8 × 10^3^.

**Fig 5:**
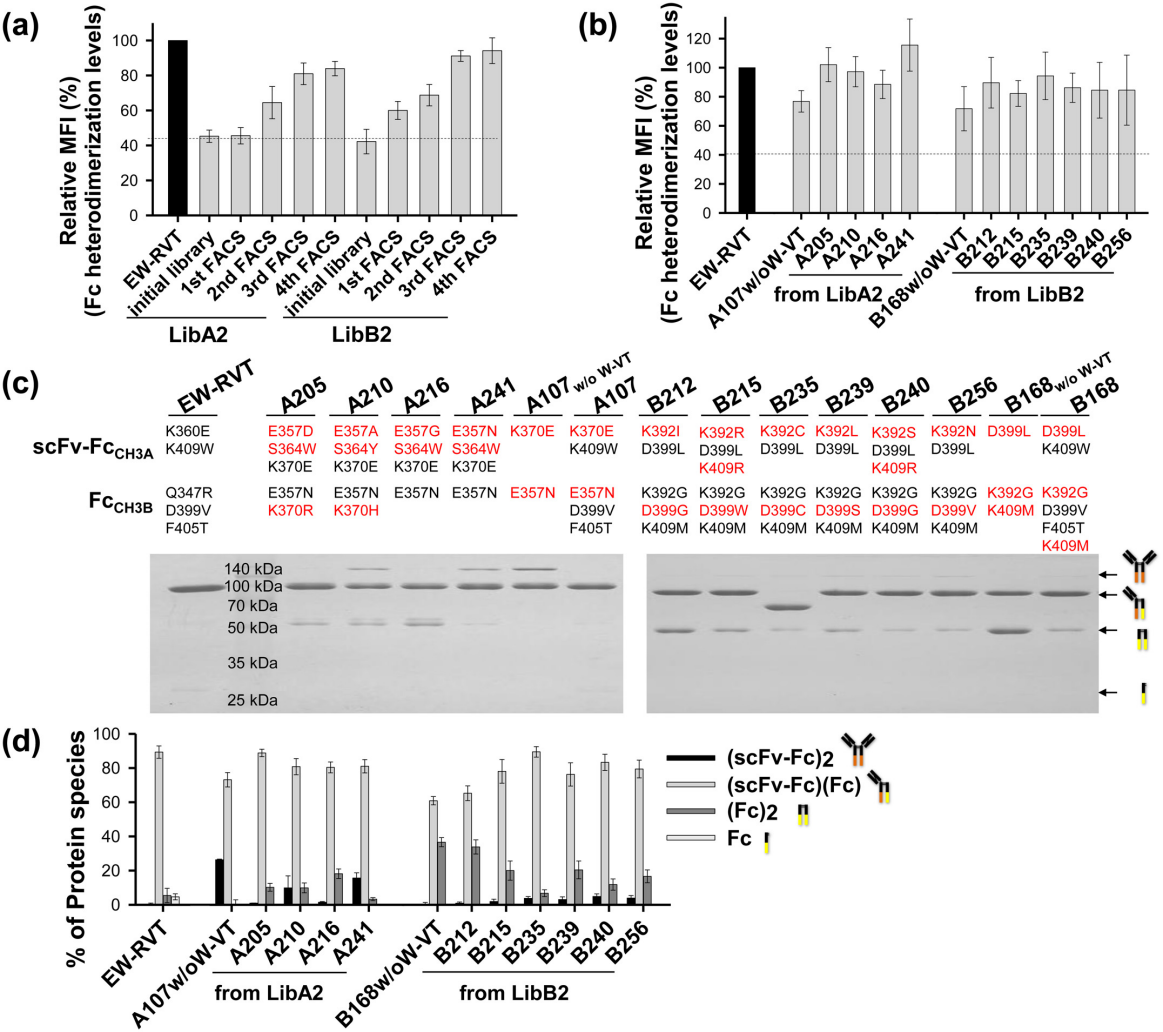
Isolation and characterization of the heterodimeric Fc variants from the LibA2 and LibB2 libraries. (a) Enrichment profiles of diploid cells with higher Fc heterodimerization levels during 4 sequential rounds of FACS with each library, as shown by the relative MFI (%) of each sample compared with that of the EW-RVT Fc variant (set as 100%). (b) Comparison of the heterodimerization levels of isolated Fc variants on the yeast cell surface expressed as a percentage of the MFI relative to that of the EW-RVT Fc variant (set as 100%). (c) SDS-PAGE analyses under non-reducing conditions of the purified coexpressed scFv-Fc_CH3A_/Fc_CH3B_ proteins (5 μg) carrying the indicated CH3 variant pair. The newly introduced mutations in the CH3A or CH3B domain of isolated the Fc variants are shown in red font. The EW-RVT, A107_w/o W-VT_, and B168_w/o W-VT_ variants are included for comparison. (d) Heterodimer yield of the indicated scFv-Fc_CH3A_/Fc_CH3B_ proteins, estimated as described in Fig 3e.

As with LibA1 and LibB1 (Fig 3a), we carried out four rounds of FACS to enrich the library pools for variants displaying a high amount of Fc heterodimer on the cell surface (Fig 5a). Analysis of more than 40 clones randomly chosen from the final sorted pool yielded four unique Fc variants (A205, A210, A216, and A241) from LibA2 and six unique Fc variants (B212, B215, B235, B239, B240, and B256) from LibB2 (Table 2) that showed much higher levels of surface-displayed Fc heterodimer than the parent A107_w/o W-VT_ and B168_w/o W-VT_ variants (Fig 5b). Again, the heterodimerization yields of all the isolated clones were assessed by SDS-PAGE analysis of the two scFv-Fc_CH3A_/Fc_CH3B_ variant protein coexpressed in HEK293F cells (Fig 5c) [7, 8]. The new Fc variants showed higher heterodimerization yields than the parent A107_w/o W-VT_ and B168_w/o W-VT_ variants, and the A205 (~89%) and B235 (~89%) variants showed the highest yields among the variants from LibA2 and LibB2, respectively (Fig 5d and Tables 1 and 2).

**Fig 6:**
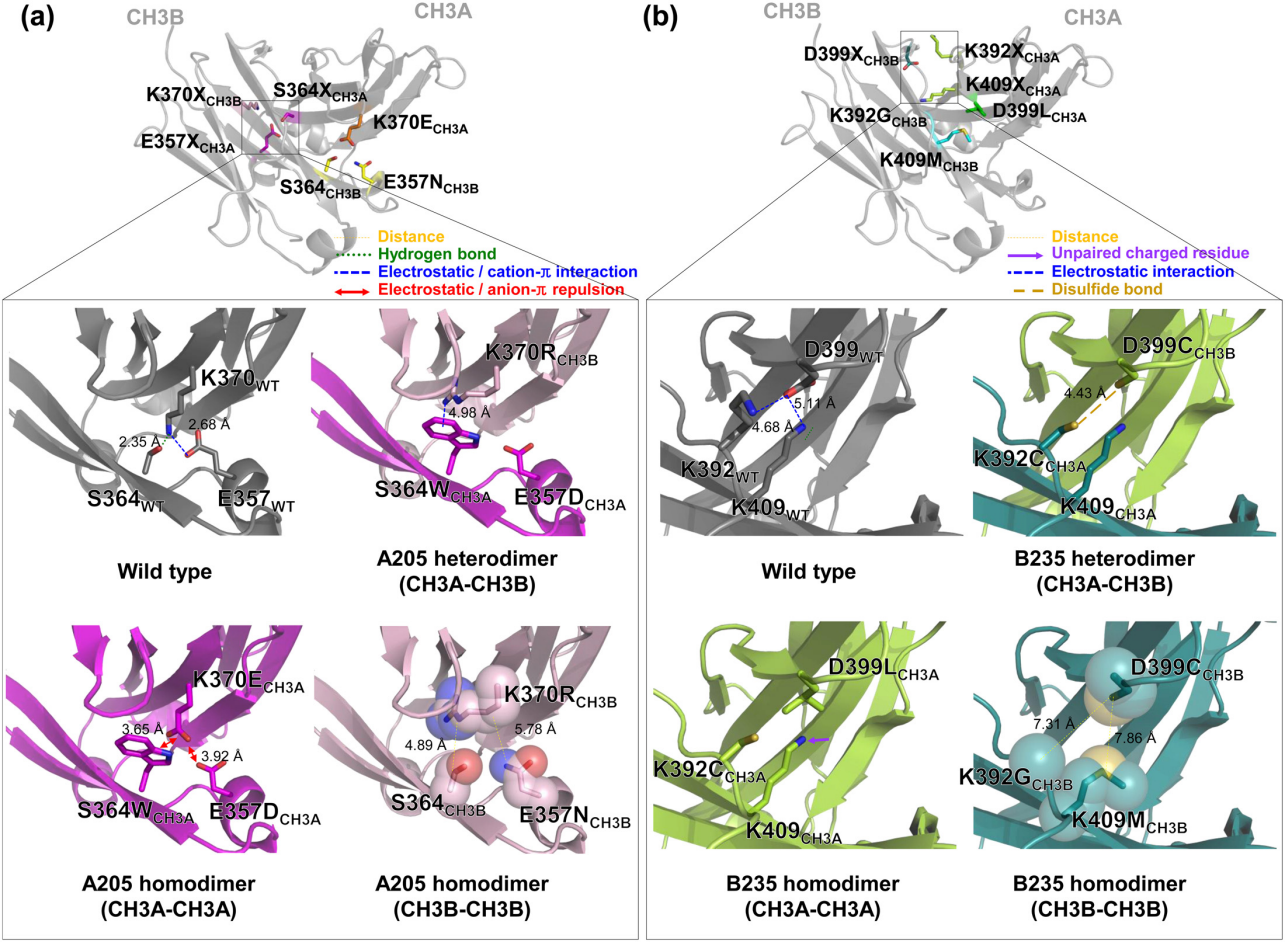
Modeled CH3 domain interface structures of the A205 (a) and B235 (b) variants based on the crystal structure of the EW-RVT variant (PDB code 4X98). Each upper panel shows the overall CH3A-CH3B heterodimer structure of the A107_w/o W-VT_ (a) and B168_w/o W-VT_ (b) variants to show the locations of the targeted mutation sites in LibA2 (a) and LibB2 (b) (as highlighted in the squares) relative to the parent K370E_CH3A_-E357N_CH3B_ mutation pair in A107_w/o W-VT_ (a) and the D399L_CH3A_-K392G/K409M_CH3B_ mutation pair in B168_w/o W-VT_ (b), respectively. The lower panels show a close-up view of the newly introduced mutation pair in A205 (E357D/S364W_CH3A_-K370R_CH3B_) (a) and B235 (K392C_CH3A_-D399C_CH3B_) (b) in a CH3A-CH3B heterodimer, CH3A-CH3A homodimer, and CH3B-CH3B homodimer, compared to the wild type CH3 homodimer. Details are described in the text.

To understand the reasons for the improved heterodimerization yields, we analyzed the modeled CH3 interfaces of the isolated heterodimeric Fc variants. Very intriguingly, most of the isolated heterodimeric Fc variants from LibA2 maintained positively charged amino acids at K370_CH3A_ (Lys, Arg, or His), but had mutations at S364_CH3A_, including amino acids with bulky aromatic groups (Trp or Tyr; Table 1). One representative variant, A205, which had the highest heterodimerization yield of ~89% (Table 1), the mutation pair seemed to contribute to heterodimerization through cation-π interactions between S364W_CH3A_-K370R_CH3B_ (A205; Fig 6a) and S364W_CH3A_-K370_CH3B_ (A216 and A241) as well as π-π interactions between S364Y_CH3A_-K370H_CH3B_ (A210) [9, 26]. CH3A-CH3A and CH3B-CH3B homodimerization of the LibA2 variants was mostly discouraged by unfavorable anion-π interactions and the hole-hole interface, respectively (Fig 6a and Table 1). For LibB2 variants, the newly introduced mutations seemed to favor heterodimerization due to cation-π interactions between the positively charged residue at 409_CH3A_ and the residues (Trp or Phe) at 405_CH3B_, while CH3A-CH3A homodimerization was disfavored by unpaired charged residues, and CH3B-CH3B homodimerization was disfavored due to the hole-hole interface (Fig 6b). Notably, the B235 variant possessed the K392C_CH3A_-D399C_CH3B_ mutation pair, which is 4.43 Å apart, an optimal distance for disulfide bonding (Fig 6b) [27]. Thus, the relatively high heterodimerization yield (~89%) for the B235 variant could be attributed to asymmetric disulfide bonding at the CH3 interface.

### Biochemical characterization of purified heterodimeric Fc variants

**Fig 7:**
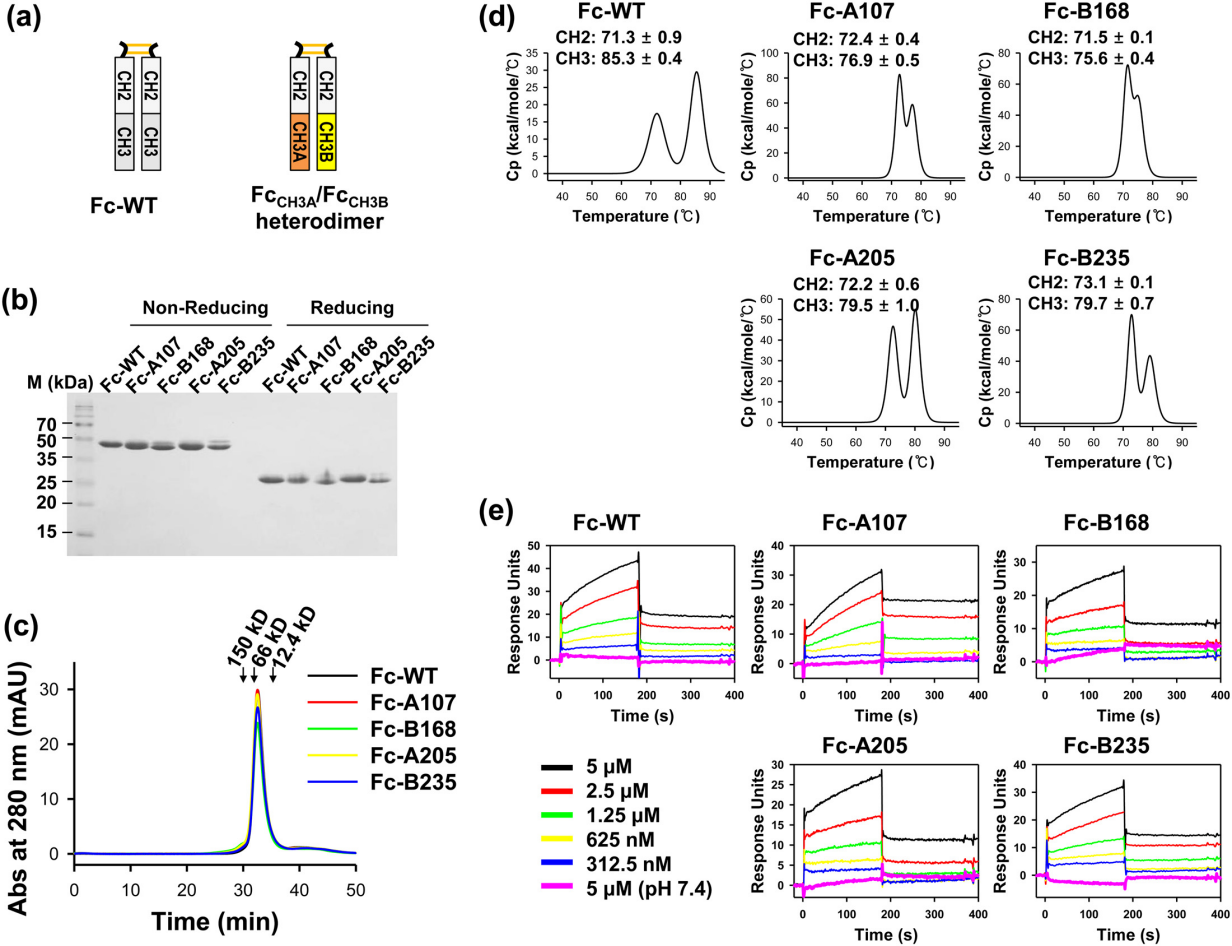
Biochemical characterization of the heterodimeric Fc variants (A107, B168, A205, and B235) in the Fc_CH3A_-Fc_CH3B_ format compared to the wild-type (WT) Fc homodimer. (a) Schematic drawing of the Fc dimer, assembled by coexpression of two Fc fragments carrying the WT CH3 pair (Fc-WT), A107 (Fc-A107), B168 (Fc-B168), A205 (Fc-A205), or B235 (Fc-B235) CH3A-CH3B variant pair. (b–e) Reducing and non-reducing SDS-PAGE analysis (b), SEC elution profiles (c), DSC thermograms (d), and SPR sensorgrams showing pH-dependent kinetic interactions with human FcRn (e) of the purified Fc-WT, Fc-A107, Fc-B168, Fc-A205, and Fc-B235 proteins. In (c), arrows indicate the molecular weight standards. In (d), the solid line is the best fit of the DSC thermogram to a 2-state transition model, and the *T*_m_ values obtained by fitting the experimental thermogram are shown in each panel. In (e), the serially diluted Fc proteins were applied to a chip coated with human FcRn at approximately 1,000 response units. Quantitative interaction parameters are shown in S2 Table.

To characterize the biochemical properties of the isolated Fc variants in an Fc_CH3A_-Fc_CH3B_ format, representative Fc variants (A107, B168, A205, and B235) were reformatted into 2 Fc fragments, i.e., hinge-CH2-CH3A × hinge-CH2-CH3B (Fc_CH3A_/Fc_CH3B_; Fig 7a) [8, 18], generating Fc-A107, Fc-B168, Fc-A205, and Fc-B235 variants. HEK293F cells were cotransfected with two plasmids carrying an Fc_CH3A_ and Fc_CH3B_ variant pair, and coexpression of these proteins yielded dimeric Fc proteins (Fig 7b) with purification yields comparable to that of WT Fc (Tables 1 and 2). Size exclusion chromatography (SEC) analysis of the purified heterodimeric Fc proteins showed a monodisperse peak of the expected molecular size (~51 kDa; Fig 7c), indicative of no non-native oligomers in the Fc protein preparations. Thermal stability analysis of heterodimeric Fc proteins was performed using differential scanning calorimetry (DSC; Fig 7d). Consistent with previous reports, the thermogram of Fc-WT exhibited two distinguishable thermal transitions (*T*_m_) at approximately 71.3°C and 85.3°C (Fig 7d), which can be attributed to the thermal unfolding of the CH2 and CH3 domains, respectively [15, 18]. The CH2 domain of the Fc heterodimer variants exhibited comparable *T*_m_ to that of Fc-WT. However, the *T*_m_ for the CH3 domain of the Fc variants was 5–10°C lower than that of Fc-WT, as has been reported for other Fc heterodimer variants, such as KiH [11] and EW-RVT [8]. Notably, however, the *T*_m_ values for the CH3 domains of Fc-A205 (~79.5°C) and Fc-B235 (~79.7°C) were ~2°C higher than those of the initially isolated variants Fc-A107 (~76.9°C) and Fc-B168 (~75.6°C; Fig 4d) as well as the previously reported variants Fc-EW-RVT (~77.4°C) [8, 18] and Fc-KiH (~76.2°C) [8, 11].

The pH-dependent interaction between the Fc regions of IgG antibody with the neonatal Fc receptor (FcRn) in endosomes is critical for prolonging serum half-life [28]. We determined the pH-dependent binding of the purified Fc variants to FcRn by surface plasmon resonance (SPR). Like the Fc-WT protein, all four Fc variants showed strong binding to FcRn at pH 6.0, but no substantial binding at pH 7.4 (Fig 7e), which is typical of IgG1 Fc [28]. The kinetic parameters for the binding of the Fc variants to FcRn at pH 6.0 were similar to that of Fc-WT (S1 Table). This result suggested that the newly introduced mutations at the CH3 interface do not significantly alter the structure of the Fc regions.

## Discussion

The Fc heterodimer is a very useful platform for the construction of bispecific antibodies in an IgG-like format with natural IgG properties. Until now, most Fc heterodimers have been generated by structure-guided rational design [3, 4, 9]. Here, we showed that heterodimeric Fc variants can be engineered by directed evolution approach combined with screening by yeast surface display. We developed a combinatorial heterodimeric Fc library engineering system using yeast surface display and successfully isolated heterodimeric Fc variants with high heterodimerization yields through FACS-mediated high-throughput screening.

Phage display has been used to isolate more stable KiH Fc heterodimers by coexpressing a CH3A knob mutant (T366W_CH3A_) with a CH3B hole mutant library containing three randomized residues [11]. The CH3A knob mutant was expressed as a secreted form to be displayed on the phage surface when it stably heterodimerized with the CH3B hole mutant expressed as a fusion to the M13 gene III [11]. However, this phage display system cannot be used to construct combinatorial heterodimeric CH3 libraries with mutations in both CH3A and CH3B because the proteins are expressed in a single bacterial cell. In similar approaches using yeast surface display, two proteins have been coexpressed, one in a surface-anchored form and the other in a secreted form, in a single haploid yeast cell line, and the proteins formed heterodimeric protein-protein interactions on the cell surface [29–31]. However, such a co-display system using a single haploid yeast cell line only allows display of one protein while the other partner protein is fixed [29–31]. Thus, this approach is also limited for combinatorial library construction with two separate protein libraries.

Yeast mating is a very powerful approach for constructing combinatorial libraries using two haploid cell lines of opposite mating types carrying two distinct libraries [32]. Taking advantage of yeast mating, combinatorial Fab antibody libraries have been constructed using two haploid yeast cell lines carrying heavy chain and light chain libraries [20–22]. However, there have been no reports of heterodimeric proteins engineered by constructing combinatorial libraries using two haploid yeasts each carrying a different protein library. In this study, we mated two haploid yeasts, one displayed an Fc_CH3A_ library on the cell surface and the other secreted an Fc_CH3B_ library, to construct a combinatorial heterodimeric Fc library. In the mated cells, the secreted Fc_CH3B_ was either released by the cells or displayed on the cell surface due to heterodimerization with the displayed Fc_CH3A_ (Fig 1). Accordingly, detection of secreted Fc_CH3B_ anchored on the cell surface by immunofluorescence allowed semi-quantitative screening of heterodimeric Fc variants with high heterodimerization yields. Thus, displaying the heterodimeric Fc library on diploid cells provides a genotype-phenotype link for high-throughput screening and easy recovery of paired Fc variants by colony PCR [20, 21]. Screening of the libraries by double fluorescence labeling of surface displayed Fc_CH3A_ for the surface expression levels and secreted Fc_CH3B_ for the heterodimerization levels isolated high-yield heterodimeric Fc variants with similar expression levels (Tables 1 and 2). The heterodimerization levels estimated from the amount of surface-displayed Fc_CH3B_ were largely in agreement with those assessed by the scFv-Fc_CH3A_/Fc_CH3B_ system in mammalian HEK293F cells (Figs 3 and 5). This suggests that the heterodimeric Fc is only displayed on the cell surface due to interactions between the CH3 variant pairs during secretion, mimicking Fc heterodimerization in mammalian cells [2].

Directed evolution of heterodimeric Fc variants by simultaneous mutation of the homodimer-favoring electrostatic interaction pairs K370-E357 and D399-K392/K409 in both the CH3A and CH3B domains generated heterodimeric Fc variants with unexpected CH3 interface mutation pairs (Tables 1 and 2), which might not have been generated through structure-guided design approaches. Structure-based rational design methods for engineering heterodimeric Fc via electrostatic interaction can be largely classified into two types [9]: 1) substitution of the conserved symmetric electrostatic interactions at the CH3 interface with asymmetric electrostatic interactions, like K392D/K409D_CH3A_-E356K/D399K_CH3B_ in the DD-KK Fc variant [7, 17] and 2) replacement of the conserved, symmetric electrostatic interactions at the buried interface of the CH3 domains with asymmetric hydrophobic interactions like K409W_CH3A_-D399V/F405T_CH3B_ in the W-VT Fc variant ([8, 18]. Although some variants had CH3 interface mutation pairs favoring heterodimerization via asymmetric steric complementarity or electrostatic interactions, in many variants, heterodimerization appeared to be driven by hydrogen bonding (e.g., A107 and A146) or cation-π interactions between the electron-rich aromatic ring of amino acids such as Trp, Tyr, and Phe and adjacent positively charged amino acids such as Arg and Lys [26] (e.g., A205, A216, A241, B212, B215, B240, and B256; Tables 1 and 2).

Another benefit of Fc engineering using yeast surface display is the ability to select correctly folded Fc variants without compromising thermodynamic stability due to the correlation between protein stability and expression, which is mediated by the quality control system in the endoplasmic reticulum (ER) of yeast cells [19, 33]. Yeast surface display has been used to engineer the Fc region of human IgG1 to increase its thermal stability [34]. In our study, the isolated heterodimeric Fc variants maintained pH-dependent FcRn binding and possessed slightly improved thermal stability in the CH3 domain compared to previously reported Fc variants derived from structure-based design.

In conclusion, our approach of combinatorial heterodimeric Fc library construction on the yeast cell surface via mating and high-throughput screening using FACS enabled us to generate Fc heterodimer variants with previously inaccessible heterodimer-favoring CH3 mutation pairs. Therefore, our studies provide a new platform for engineering Fc heterodimer variants with improved heterodimerization yields and biophysical properties. Furthermore, our approach could be used to engineer other heterodimeric protein-protein interactions through directed evolution combined with yeast surface display.

## Supporting Information

S1 Fig Schematic presentation of yeast expression plasmids, pCTCON-displayed Fc_CH3A_ and pSEC2-secreted Fc_CH3B_. (DOCX)

S2 Fig Evaluation of heterodimerization yields of the representative heterodimeric Fc variants (A107, B168, A205, and B235) depending on the co-transfected molar rations of two DNAs encoding scFv-Fc_CH3A_ and Fc_CH3B_ at indicated ratio in the panel. (DOCX)

S1 Table The kinetic parameters for the interactions of Fc proteins with FcRn proteins, determined by SPR^a^
(DOCX)

S2 Table Primer sequences used for Fc haploid library construction. (DOCX)
