# Supplementary material for: Engineering of Immunoglobulin Fc Heterodimers Using Yeast Surface-Displayed Combinatorial Fc Library Screening
Source: PLoS One. 2015 Dec 16;10(12):e0145349. doi: 10.1371/journal.pone.0145349 (PMC4682967; doi:10.1371/journal.pone.0145349)
Supplement: S1 Fig — (DOCX) [file pone.0145349.s001.docx]

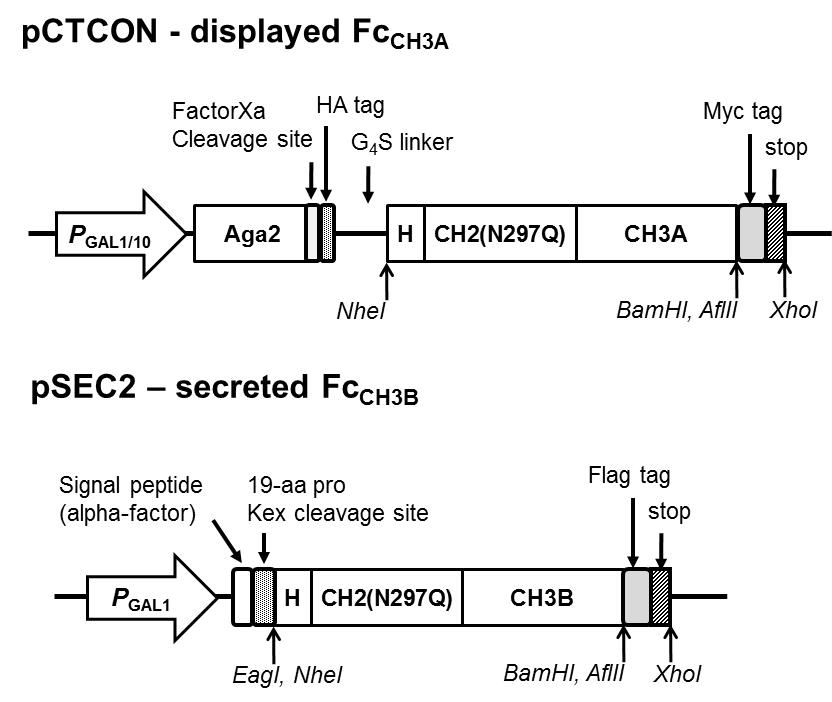


**S1 Fig.** Schematic presentation of yeast expression plasmids, pCTCON-displayed Fc_CH3A_ and pSEC2-secreted Fc_CH3B_. The plasmid pCTCON-displayed Fc (*TRP1* marker) was designed based on pCTCON to express one Fc variant (hinge-CH2-CH3A) as its N-terminal fusion to Aga2 to be anchored on the yeast cell surface and C-terminal Myc tag under the control of *GAL1-10* promoter. The plasmid pSEC2-secreted Fc was designed based on pRS316 (*URA3* maker) to secrete another Fc variant (hinge-CH2-CH3B) with its C-terminal Flag tag under *GAL1* promoter without Aga2 fusion (Supplementary Fig. S1). The Fc region of human IgG1 includes the hinge-CH2-CH3 regions (residues 225-447 in EU number). In both plasmids, the bottom hinge region sequence (THTCPPCP) of Fc was modified by substitution of Cys with Ser (THTSPPSP) to avoid Fc homodimerization by the hinge region disulfide bond. Further, the N-glycosylation site at Asn297 of Fc regions was substituted with Gln (N297Q) to exclude the yeast hypermannosylation. *P_GAL1/10_*, GAL1/10 promoter; *P_GAL1_*, GAL1 promoter; *H*, human IgG1 hinge sequence; *CH3A and CH3B*, human IgG1 CH3 variant sequence; *stop*, translational stop sequence.
