# Supplementary material for: Engineering of Immunoglobulin Fc Heterodimers Using Yeast Surface-Displayed Combinatorial Fc Library Screening
Source: PLoS One. 2015 Dec 16;10(12):e0145349. doi: 10.1371/journal.pone.0145349 (PMC4682967; doi:10.1371/journal.pone.0145349)
Supplement: S2 Fig — (DOCX) [file pone.0145349.s002.docx]

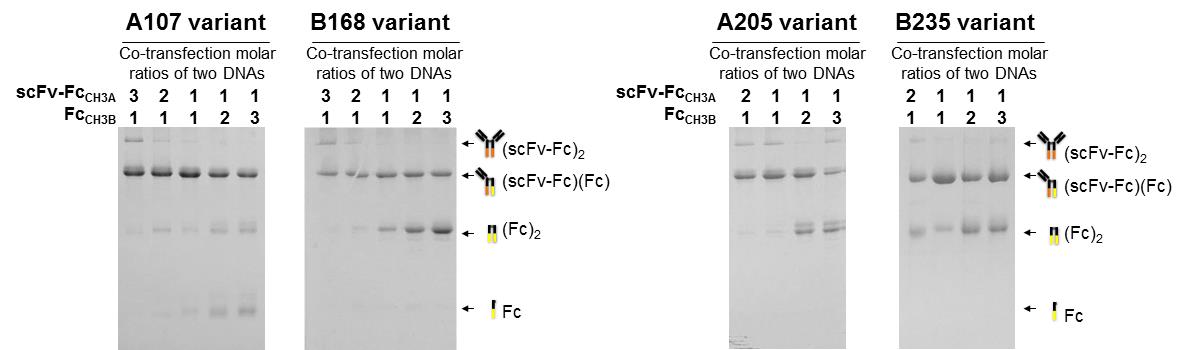


**S2 Fig.** Evaluation of heterodimerization yields of the representative heterodimeric Fc variants (A107, B168, A205, and B235) depending on the co-transfected molar rations of two DNAs encoding scFv-Fc_CH3A_ and Fc_CH3B_ at indicated ratio in the panel. The purified proteins (each 5μg) were analyzed by SDS-PAGE under non-reducing conditions. The arrows indicate the assembled scFv-Fc_CH3A_ homodimer (~150 kDa), scFv-Fc_CH3A_/Fc_CH3B_ heterodimer (~78 kDa), Fc_CH3B_ homodimer (~53 kDa), and unassembled Fc_CH3B_ monomeric species (~ 27 kDa).
