# Supplementary material for: Engineering of Immunoglobulin Fc Heterodimers Using Yeast Surface-Displayed Combinatorial Fc Library Screening
Source: PLoS One. 2015 Dec 16;10(12):e0145349. doi: 10.1371/journal.pone.0145349 (PMC4682967; doi:10.1371/journal.pone.0145349)
Supplement: S1 Table — (DOCX) [file pone.0145349.s003.docx]

**S1 Table.** The kinetic parameters for the interactions of Fc proteins with FcRn proteins, determined by SPR^a^

| **Proteins** | **Fc proteins** | ***k*_on_ (M^-1^s^-1^)** | ***k*_off_ (s^-1^)** | ***K*_D_ (M)** |
| --- | --- | --- | --- | --- |
| FcRn  (pH 6.0) | Fc-WT | 1.64±0.05×10^3^ | 5.83±0.46×10^-5^ | 3.56±0.39×10^-8^ |
|  | Fc-A107 | 1.58±0.03×10^3^ | 1.02±0.69×10^-4^ | 6.50±0.45×10^-8^ |
|  | Fc-B168 | 1.09±0.01×10^3^ | 1.67±0.39×10^-5^ | 1.75±0.15×10^-8^ |
|  | Fc-A205 | 1.13±0.01×10^3^ | 1.11±0.57×10^-4^ | 1.18±0.39×10^-8^ |
|  | Fc-B235 | 1.58±0.02×10^3^ | 8.00±0.57×10^-5^ | 5.06±0.43×10^-8^ |

^a^ Each value represents the mean ± SD of two independent experiments. In each experiment, at least 5 data sets were used in the determination of the kinetic constants. The representative sensograms are shown in Fig. 7e.
