# Supplementary material for: Engineering of Immunoglobulin Fc Heterodimers Using Yeast Surface-Displayed Combinatorial Fc Library Screening
Source: PLoS One. 2015 Dec 16;10(12):e0145349. doi: 10.1371/journal.pone.0145349 (PMC4682967; doi:10.1371/journal.pone.0145349)
Supplement: S2 Table — (DOCX) [file pone.0145349.s004.docx]

**S2 Table.** Primer sequences used for Fc haploid library construction.

| **Library** | | **Aim** | **Direction** | **Nucleotide sequences (5’-3’)** |
| --- | --- | --- | --- | --- |
| **LibA1** | **Display Fc**  **(CH3A)** | Amplifying Fc fragment 1 | Forward | GTTCCAGACTACGCTCTGCAGG |
|  |  | Amplifying Fc fragment 1 | Reverse | GACCAGGCAGGTCAGGCTGA |
|  |  | Amplifying Fc fragment 2 | Forward | TCAGCCTGACCTGCCTGGTC**DNB**GGCTTCTATCCCAGCGACATCG |
|  |  | Amplifying Fc fragment 2 | Reverse | TCGATTTTGTTACATCTACACTGTTGTTATCAGAT |
|  | **Secretion Fc**  **(CH3B)** | Amplifying Fc fragment 1 | Forward | CTCAACCGGTTATTTCTACTACCGTC |
|  |  | Amplifying Fc fragment 1 | Reverse | ATCCCGGGATGGGGGCAG |
|  |  | Amplifying Fc fragment 2 | Forward | CTGCCCCCATCCCGGGAT**DNB**CTGACCAAGAACCAGGTC**DNB**CTGACCTGCCTGGTCAAAGGC |
|  |  | Amplifying Fc fragment 2 | Reverse | GAACAAAGTCGATTTTGTTACATCTACACTGTT |
| **LibB1** | **Display Fc**  **(CH3A)** | Amplifying Fc fragment 1 | Forward | GTTCCAGACTACGCTCTGCAGG |
|  |  | Amplifying Fc fragment 1 | Reverse | CAGCACGGGAGGCGTGGT |
|  |  | Amplifying Fc fragment 2 | Forward | ACCACGCCTCCCGTGCTG**DNB**TCCGACGGCTCCTTCTTCCTC |
|  |  | Amplifying Fc fragment 2 | Reverse | TCGATTTTGTTACATCTACACTGTTGTTATCAGAT |
|  | **Secretion Fc**  **(CH3B)** | Amplifying Fc fragment 1 | Forward | CTCAACCGGTTATTTCTACTACCGTC |
|  |  | Amplifying Fc fragment 1 | Reverse | AGGAGCCGTCGGACACCAGCACGGGAGGCGTGGT**VNH**GTAGTTGTTCTCCGGCTGCCC |
|  |  | Amplifying Fc fragment 2 | Forward | CTGGTGTCCGACGGCTCCTTCACCCTCTACAGC**DNB**CTCACCGTGGACAAGAGCAGG |
|  |  | Amplifying Fc fragment 2 | Reverse | GAACAAAGTCGATTTTGTTACATCTACACTGTT |
| **LibA2** | **Display Fc**  **(CH3A)** | Amplifying Fc fragment 1 | Forward | GTTCCAGACTACGCTCTGCAGG |
|  |  | Amplifying Fc fragment 1 | Reverse | ATCCCGGGATGGGGGCAG |
|  |  | Amplifying Fc fragment 2 | Forward | CTGCCCCCATCCCGGGAT**NNK**CTGACCAAGAACCAGGTC**NNK**CTGACCTGCCTGGTCGAGGGC |
|  |  | Amplifying Fc fragment 2 | Reverse | TCGATTTTGTTACATCTACACTGTTGTTATCAGAT |
|  | **Secretion Fc**  **(CH3B)** | Amplifying Fc fragment 1 | Forward | CTCAACCGGTTATTTCTACTACCGTC |
|  |  | Amplifying Fc fragment 1 | Reverse | GACCAGGCAGGTCAGGCTGA |
|  |  | Amplifying Fc fragment 2 | Forward | TCAGCCTGACCTGCCTGGTC**NNK**GGCTTCTATCCCAGCGACATCG |
|  |  | Amplifying Fc fragment 2 | Reverse | GAACAAAGTCGATTTTGTTACATCTACACTGTT |
| **LibB2** | **Display Fc**  **(CH3A)** | Amplifying Fc fragment 1 | Forward | GTTCCAGACTACGCTCTGCAGG |
|  |  | Amplifying Fc fragment 1 | Reverse | AGGAGCCGTCGGACAACAGCACGGGAGGCGTGGT**MNN**GTAGTTGTTCTCCGGCTGCCC |
|  |  | Amplifying Fc fragment 2 | Forward | CTGTTGTCCGACGGCTCCTTCTTCCTCTACAGC**NNK**CTCACCGTGGACAAGAGCAGG |
|  |  | Amplifying Fc fragment 2 | Reverse | TCGATTTTGTTACATCTACACTGTTGTTATCAGAT |
|  | **Secretion Fc**  **(CH3B)** | Amplifying Fc fragment 1 | Forward | CTCAACCGGTTATTTCTACTACCGTC |
|  |  | Amplifying Fc fragment 1 | Reverse | CAGCACGGGAGGCGTGGT |
|  |  | Amplifying Fc fragment 2 | Forward | ACCACGCCTCCCGTGCTG**NNK**TCCGACGGCTCCTTCTTCCTC |
|  |  | Amplifying Fc fragment 2 | Reverse | GAACAAAGTCGATTTTGTTACATCTACACTGTT |

Bold: Diversification region.
